# Evaluation of the Inflammatory Response in Macrophages Stimulated with Exosomes Secreted by *Mycobacterium avium*-Infected Macrophages

**DOI:** 10.1155/2015/658421

**Published:** 2015-03-16

**Authors:** Jianjun Wang, Yongliang Yao, Jing Xiong, Jianhong Wu, Xin Tang, Guangxin Li

**Affiliations:** ^1^Department of Clinical Laboratory, Kunshan First People's Hospital Affiliated to Jiangsu University, Kunshan 215300, China; ^2^Department of Ophthalmology, Xiangya Hospital, Central South University, Changsha, Hunan 410078, China; ^3^Department of Pathology, ChongQing Cancer Institute, Chongqing 400030, China

## Abstract

Exosomes secreted from *Mycobacterium avium*-infected macrophages contain numerous antigens of both *M. avium* and the host cell and are involved in the induction and expression of the inflammatory responses in macrophages. The interaction between exosomes secreted from *M. avium*-infected macrophages and macrophage phagocytosis, cytokine secretion, immunostimulation, and apoptosis was analyzed. Upon stimulation with exosomes secreted from *M. avium*-infected macrophages, the phagocytosis of dextran by treated macrophages was increased. Furthermore, the expression of CD40, CD80, CD81, CD86, HLA-DR, and most notably CD195 was enhanced. Additionally, the secretion of IL-6, IL-8, IL-10, IFN-*γ*, and TNF-*α* was increased by stimulated macrophages. Exosome stimulation did not induce macrophage apoptosis when compared with macrophages infected with *M. avium*. Caspase expression, including that of caspases 3, 6, and 8, was also not altered in exosome stimulated macrophages. Thus exosomes trigger the inflammatory response in macrophages owing to the presence of bacterial antigens but have no effect on macrophage viability.

## 1. Introduction

*Mycobacterium tuberculosis* infection is a severe global health problem, and China is among the worst affected countries [1, 2]. The immune response to* M. tuberculosis* infection requires the activation of alveolar macrophages and the development of a Th1-type CD4^+^ T cell response, leading to the formation of lung granulomas [3]. Alveolar macrophages, as the main resident immune cells in the lung, are activated to produce cytokines including interferon *γ* (IFN-*γ*), tumor necrosis factor *α* (TNF-*α*), interleukin 1 (IL-1), IL-10, and IL-12, which regulate the production of nitric oxide and reactive oxygen species to kill or inhibit mycobacteria [4, 5].* Mycobacterium avium* shares with* M. tuberculosis* a slow growth rate and an ability to generate granulomas and is itself pathogenic [6–9].* M. avium*- and* M. tuberculosis*-containing phagosomes share important features including restricted fusing with endosomal/lysosomal compartments [10–12] and impaired acidification [13, 14].

Exosomes are small membranous vesicles generated by inward budding of late endosomes, resulting in the formation of multivesicular bodies in the cell cytosol. Exosomes can be derived from B cells and antigen presenting cells such as macrophages, dendritic cells (DCs), and natural killer cells, which are enriched in proteins of the tetraspanin family including CD63 and CD81 [15] and molecules involved in antigen presentation to sensitized T cells (CD80, CD86, and MHC-II) [16]. Thus exosomes play pivotal roles in both physiological crosstalk between cells and disease pathogenesis.

In general, exosomes act as molecular carriers during immune cell-cell communication [17]. However, recent studies also show that exosomes carrying tumor antigens promoted antigen-specific T cell activation and tumor rejection in vivo [18]. Knowledge of the protein composition of exosomes suggests further functions for these extracellular vesicles, for instance, exosomes released from* Mycobacterium*-infected macrophages carry mycobacterial antigens including lipoprotein and lipoarabinomannan [19]. Additionally, Hsp-70 in exosomes induces a proinflammatory response [20] and exosomes containing glycopeptidolipids of* M. avium* transfer them from infected to noninfected macrophages, resulting in a toll-like receptor-dependent proinflammatory response [21].

Exosomes can not only induce inflammatory responses, but could also modulate immune responses, including both immune stimulation and immune suppression [22]. Recent reports have also shown that exosomes containing microbial antigens were able to protect against microorganism infection. Studies by Colino and Snapper indicated that injecting mice with exosomes containing the capsular polysaccharide type 14 cross-reactive antigen of* Streptococcus pneumoniae* would induce a protective antibody response to resist* S. pneumoniae* [23]. Similarly, treating mice with exosomes derived from DCs pulsed with toxoplasma antigens was also shown to protect the mice against subsequent toxoplasma infection [24]. At present, there are approximately 29 kinds of* M. tuberculosis* proteins found in exosomes released from CFP-treated J774 cells, the majority of which were also present in exosomes isolated from* M. tuberculosis*-infected cells. The exosomes from CFP-treated J774 cells could promote macrophage and DC activation as well as activation of naïve T cells in vivo. This suggests that exosomes containing* M. tuberculosis* antigens may be alternative approach to developing a novel tuberculosis vaccine [25].

In this study, we analyzed the inflammatory response and apoptosis induced by exosomes secreted from* M. avium*-infected macrophages.

## 2. Materials and Methods

### 2.1. Macrophage Culture

The human acute monocytic leukemia cell line THP-1 (ATCC TIB-202) was purchased from American Type Culture Collection. Cells were cultured in wells or flasks at 37°C under 5% CO_2_, in RPMI 1640-GlutaMAX (HyClone Laboratories, GE Healthcare Lifesciences, Logan, UT, USA) containing 10% (v/v) fetal bovine serum (HyClone), 100 U/mL penicillin, 0.1 mg/mL streptomycin, and 0.25 *μ*g/mL amphotericin B. Differentiation of THP1 cells into macrophage-like cells was induced by stimulation with 0.1 mmol/L phorbol 12-myristate 13-acetate (Sigma-Aldrich, St. Louis, MO, USA) for 24 h. Mycoplasma contamination was detected using PlasmoTest (Invivogen) and mycoplasma-free cells were used in the downstream experiments.

### 2.2. *Mycobacterium avium* Culture

*Mycobacterium avium* sp.* Paratuberculosis* (referred to as* M. avium* in this paper) was obtained from the Chinese Center for Disease Control and Prevention. Bacteria were grown on Middlebrooks 7H9 plates as previously described [26] for 4 weeks, at 37°C. Colonies were harvested by scraping, with 0.9% NaCl as vehicle, and* Mycobacterium* concentrations were calculated according to the McFarland Standards method. The concentration was then adjusted to 1.5 × 10^9^ *Mycobacterium*/mL.

### 2.3. *Mycobacterium avium* Infection of Macrophages

Macrophages were cultured at 1 × 10^6^ cells per well (in 1 mL culture medium) in six-well plates and infected with* M. avium* at an MOI of 100 for 24 h, as previously described with modifications [26].* M. avium* infection rate was determined by acid-fast staining test. Culture supernatants were collected and used for cytokine analysis and exosomes isolation. The macrophages were washed with phosphate-buffered saline (PBS) and apoptosis and the expression of cell surface molecules and caspase proteins analyzed.

### 2.4. Exosome Isolation

Macrophage cell culture supernatants were centrifuged at 3,000*g* for 15 minutes to remove cells and cell debris; then supernatants were transferred to sterile tubes. The ExoQuick Exosome Precipitation Solution (System Biosciences (SBI), Mountain View, CA, USA) was added to cell culture supernatants, the tubes mixed by inverting, then refrigerated for 30 minutes. ExoQuick/biofluid mixture was centrifuged at 1,500*g* for 30 minutes; then the supernatant was aspirated. Spin-down residual ExoQuick solution was added and centrifuged at 1,500*g* for 5 minutes and all traces of fluid were removed by aspiration. Finally, exosome pellets were resuspended in 1/10 of the original volume using nuclease-free water. The exosomes pellets were mixed with 25 *μ*L of 9% sucrose containing protease inhibitors and stored at −80°C until use. All procedures were carried out at 4°C. The abbreviation (+)exosomes describes exosomes obtained from* M. avium*-infected macrophages, and the term (−)exosomes describes exosomes from noninfected macrophages. All exosomes samples were tested for endotoxin contamination using Limulus Amebocyte Lysate (LAL) test (Catalog number KT05, Houshiji, Co. Ltd.).

### 2.5. Electron Microscopy and Electrophoresis of Isolated Exosomes

Freshly isolated exosome pellets were resuspended and fixed in phosphate buffer containing 2% glutaraldehyde and then loaded on Formvar/carbon-coated electron microscopy grids. The samples were contrasted in uranyl acetate and viewed with a Hitachi H-600 transmission electron microscopy (TEM) microscope (Hitachi High-Technologies, Tokyo, Japan) at 70,000x magnification. Additionally, (+)exosomes (50 *μ*g) or (−)exosomes (50 *μ*g) samples were separated by 10% sodium dodecyl sulfate polyacrylamide gel electrophoresis (SDS-PAGE) and then compared.

### 2.6. Phagocytosis Assays

Fluorescein isothiocyanate- (FITC-) conjugated dextran (molecular mass 40 kDa, Molecular Probes, Life Technologies, Carlsbad, CA, USA) was used to determine the phagocytic function of macrophages.* M. avium-*infected or uninfected macrophages and exosome treated cells were adjusted to a concentration of 1 × 10^5^ cells in 100 *μ*L of complete RPMI 1640 medium and preincubated on ice for 30 min. Then, the above cells were incubated with 20 mg/L dextran-FITC for 30 min at 37°C or at 4°C to detect nonspecific binding. Cells were washed three times with 500 *μ*L of complete RPMI 1640 medium and fixed in 10% (vol/vol) formaldehyde-PBS. Median fluorescence intensities (MFIs) and the percentage of dextran-positive cells were determined by flow cytometry (Beckman MoFLo XDP, Beckman Coulter Inc., Indianapolis, IN, USA).

### 2.7. Flow Cytometry Analysis

Macrophages cultured in six-well plates were treated for 24 h with LPS (50 ng/mL), (−)exosomes (50 *μ*g/mL) or (+)exosomes (50 *μ*g/mL), or* M. avium* (multiplicity of infection (MOI) of 10), then harvested, and washed twice with PBS containing 0.2% BSA. Cells were then stained with fluorescein isothiocyanate- (FITC-) or phycoerythrin- (PE-) labeled monoclonal antibodies (BD, Franklin Lakes, NJ, USA) to CD25, CD32, CD40, CD80, CD81, CD86, CD163, CD195, and HLA-DR, or the appropriate isotype controls. Macrophages were washed and fixed in 10% (vol/vol) formaldehyde-PBS. Finally, analyses were performed on a Beckman MoFLo XDP flow cytometer. MFIs and the percentages of positively expressing cells were determined after subtraction of the values for the isotype controls.

### 2.8. Cytokine Quantification by ELISA

Macrophages cultured in six-well plates were treated with LPS (50 ng/mL), (−)exosomes (50 *μ*g/mL), (+)exosomes (50 *μ*g/mL), or* M. avium* (MOI of 10) for 24 h. The concentrations of IL-6, IL-8, IL-10, transforming growth factor *β*_1_ (TGF-*β*_1_), IFN-*γ*, and TNF-*α* in the cell culture supernatants of stimulated cells were analyzed by enzyme-linked immunoabsorbent assay (ELISA), according to the manufacturer's instructions (Boster Biotechnology Company, Wuhan, China). Cytokine concentrations were calculated using standard curves.

### 2.9. Western Blot Analysis

For western blotting, 50 *μ*g proteins from cell lysates, as determined by the Micro BCA Protein Assay (Merck Millipore, Billerica, MA, USA), were loaded on 10% SDS-PAGE gels, electrophoresed, and transferred onto polyvinylidene difluoride membrane (Merck Millipore). The membranes were probed for caspase-3 (R&D Systems, Minneapolis, MN, USA; 1 : 500 dilution), caspase-6 (R&D Systems, 1 : 800 dilution), and caspase-8 (R&D Systems, 1 : 400 dilution). Immunodetected protein bands were quantified with Image J software (NIH, Bethesda, MD, USA).

### 2.10. Apoptosis and Necrosis

Macrophages cultured in six-well plates were treated with LPS (50 ng/mL), (−)exosomes (50 *μ*g/mL), (+)exosomes (50 *μ*g/mL), or* M. avium* (MOI of 10) for 24 h. Apoptosis was quantitatively determined by flow cytometry using an annexin V-FITC/PI apoptosis detection kit (BD). Briefly, following treatment, cells were harvested by trypsinization, washed with PBS, and incubated with annexin V-FITC and PI at room temperature for 10 min in the dark. The stained cells were analyzed with a FACS Calibur flow cytometer and CellQuest analysis software (BD).

### 2.11. Statistical Analysis

All data are expressed as the mean ± SEM. Values were analyzed by SPSS version 16.0 software for Windows (IBM Corp., Armonk, NY, USA), and the statistical significance of differences between groups was evaluated by one-way analysis of variance. Values of *P* < 0.05 were defined as statistically significant.

## 3. Results

### 3.1. Analysis of Macrophage Exosomes

**Figure 1:**
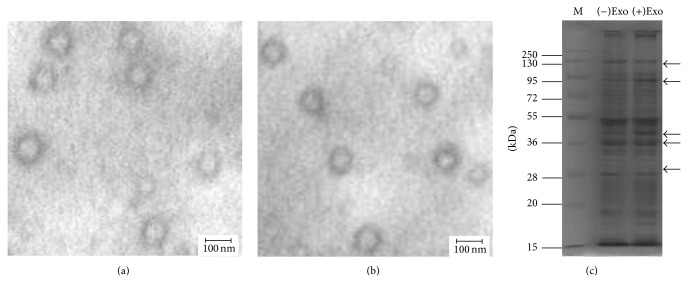
Exosomes as observed by transmission electron microscopy (TEM) and analyzed by SDS-PAGE. (a) Exosomes secreted from untreated macrophages, as viewed by TEM (70,000x magnification, scale bar: 100 nm). (b) Exosomes secreted from macrophages infected with* M. avium* viewed by TEM as per (a). (c) (−)Exosomes and (+)exosomes were analyzed by 10% SDS-PAGE. Arrows indicate variation in the concentration of specific molecular weight proteins.

TEM observation of phosphotungstic-stained, purified exosomes obtained from macrophages uninfected or infected with* M. avium* revealed a homogenous population of morphologically typical vesicles of 30 to 100 nm diameter (Figures 1(a) and 1(b)), similar in appearance and size to those in published reports [27, 28]. Additionally, electrophoresis results showed that exosomes obtained from macrophages uninfected or infected with* M. avium* were rich in proteins (Figure 1(c)), although there are obvious differences in components between (+)exosomes or (−)exosomes.

### 3.2. Characterization of Exosomes

**Figure 2:**
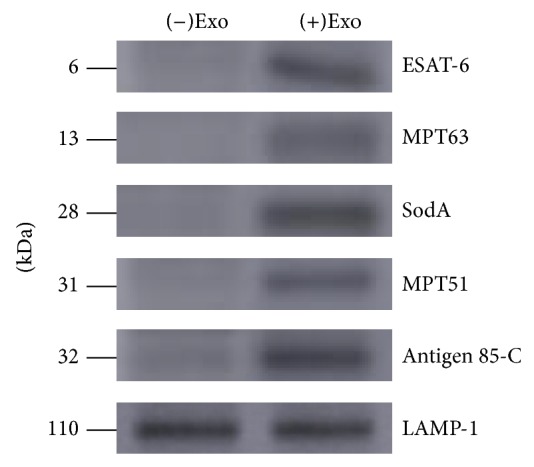
Characterization of mycobacterial proteins associated with exosomes from* M. avium*-infected macrophages. Exosomes isolated from untreated or* M. avium*-infected macrophages were analyzed by western blotting for the indicated mycobacterial proteins. Lysosomal associated membrane protein-1 (LAMP-1) was used as a positive control for both exosomes and protein loading.

To confirm that exosomes secreted from* M. avium*-infected macrophages contained proteins from* M. avium*, exosomes were probed for a subset of* M. avium* proteins by western blotting. We found that exosomes released from* M. avium*-infected macrophages, but not from uninfected cells, contained ESAT-6, MPT63, SodA, MPT51, and antigen 85 complex (antigen 85-C) (Figure 2). As expected, both exosome populations contained the host protein lysosomal associated membrane protein-1 (LAMP-1).

### 3.3. Analysis of Phagocytosis

**Figure 3:**
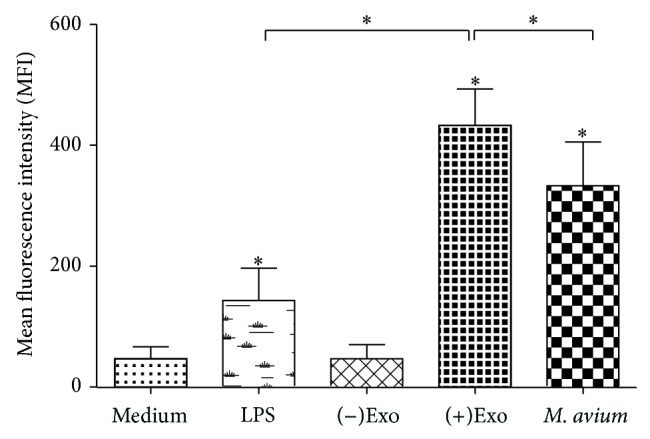
(+)Exosome-activated macrophages are characterized by increased phagocytosis. A total of 1 × 10^5^ macrophages were incubated with LPS (50 ng/mL),* M. avium* (MOI of 10), (−)exosomes or (+)exosomes (50 *μ*g/mL), or medium alone for 24 h. Data are presented as the mean ± SEM (*n* = 3 per group, ^*^ *P* < 0.05 compared with the control group).

We also investigated the phagocytic properties of macrophages preincubated with LPS, (−)exosomes, (+)exosomes, and* M. avium* compared with untreated macrophages. As shown in Figure 3, compared to medium-treated group, (−)exosomes treated macrophages did not exhibit enhanced phagocytocity, while LPS, (+)exosomes, and* M. avium*-treated macrophages showed considerable elevation. It is noteworthy that (+)exosomes are significantly stronger stimuli than LPS and* M. avium* in enhancing macrophage phagocytic activity.

### 3.4. ****(+)Exosomes Regulate the Expression of Cell Surface Molecules in Macrophages

Macrophages were treated with various stimuli including LPS (50 ng/mL), (−)exosomes (50 *μ*g/mL), (+)exosomes (50 *μ*g/mL), and* M. avium* (MOI of 10) and analyzed by flow cytometry. As evident in Table 1,* M. avium* infection increased the expression of CD32, CD40, CD80, CD81, CD86, CD163, and HLA-DR by infected macrophages, while the expression of CD25 and CD195 did not change. Notably, CD40, CD80, CD81, CD86, CD195, and HLA-DR were also significantly upregulated in (+)exosome treated macrophages compared with cells treated with (−)exosomes or medium alone.

### 3.5. ****(+)Exosome Treated Macrophages Secrete Proinflammatory Cytokines

**Figure 4:**
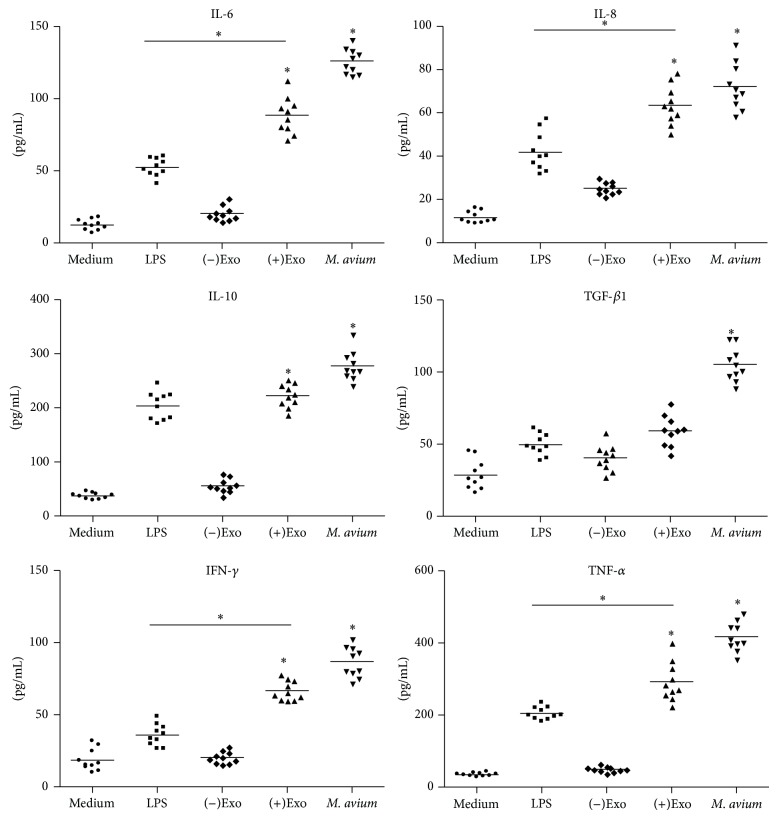
Cytokines released from macrophages treated with exosomes. A total of 1 × 10^6^ macrophages were treated with (−)exosomes (50 *μ*g/mL) or (+)exosomes (50 *μ*g/mL) and* M. avium* (MOI of 10) for 24 h. Controls included macrophages treated with 50 ng/mL LPS (squares) or incubated with medium alone. Concentrations of cytokines in the supernatants were determined by ELISA. Each symbol per condition represents the data obtained with cells from one well. Horizontal lines show the median values of 10 experiments. ^*^ *P* < 0.05 compared with medium alone (Friedman test and Dunn's multiple comparison test).

Macrophages infected with* M. avium* are able to produce a variety of cytokines including IL-6, IL-8, IFN-*γ*, and TNF-*α* in order to resist* Mycobacterium* spread. Macrophages were treated with (−)exosomes, (+)exosomes, and* M. avium* for 24 h; then IL-6, IL-8, IL-10, TGF-1*β*, IFN-*γ*, and TNF-*α* were detected in the culture supernatant by ELISA (Figure 4). Notably, IL-6, IL-8, IL-10, TGF-1*β*, IFN-*γ*, and TNF-*α* were all increased significantly in* M. avium*-infected macrophage cultures; however, the inflammatory response induced by (+)exosomes was close to that of* M. avium* infection. Therefore, the above results indicate that the inflammatory response in macrophages can be induced not only by* M. avium* infection, but also by treatment with exosomes secreted from* M. avium-*infected macrophages containing antigens of* M. avium*.

### 3.6. Caspase Proteins Expressed in Macrophages

**Figure 5:**
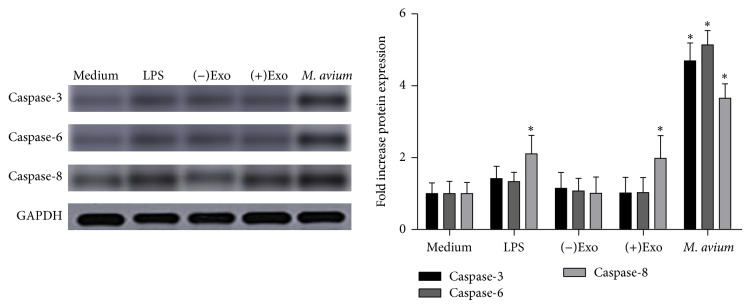
Analysis of caspase expression by western blotting. Caspases 3, 6, and 8 in macrophages treated with (−)exosomes (50 *μ*g/mL), (+)exosomes (50 *μ*g/mL), and* M. avium* (MOI of 10) were analyzed by western blotting. Controls included macrophages treated with LPS (50 ng/mL) or incubated with medium alone (mean ± SEM, 3 independent experiments). ^*^ *P* < 0.05 compared with the medium alone control.

Caspases 3, 6, and 8 are all involved at different stages of the apoptosis pathway. Caspase expression in macrophages treated with LPS, (−)exosomes, (+)exosomes, or* M. avium* was analyzed by western blotting (Figure 5), and densitometry was performed on bands using Image J software with GAPDH as a standard calibrator. Caspases 3, 6, and 8 were all demonstrated to be increased in* M. avium*-infected macrophages. However, in contrast, (+)exosomes did not influence caspase expression in treated macrophages.

### 3.7. Apoptosis of Macrophages

**Figure 6:**
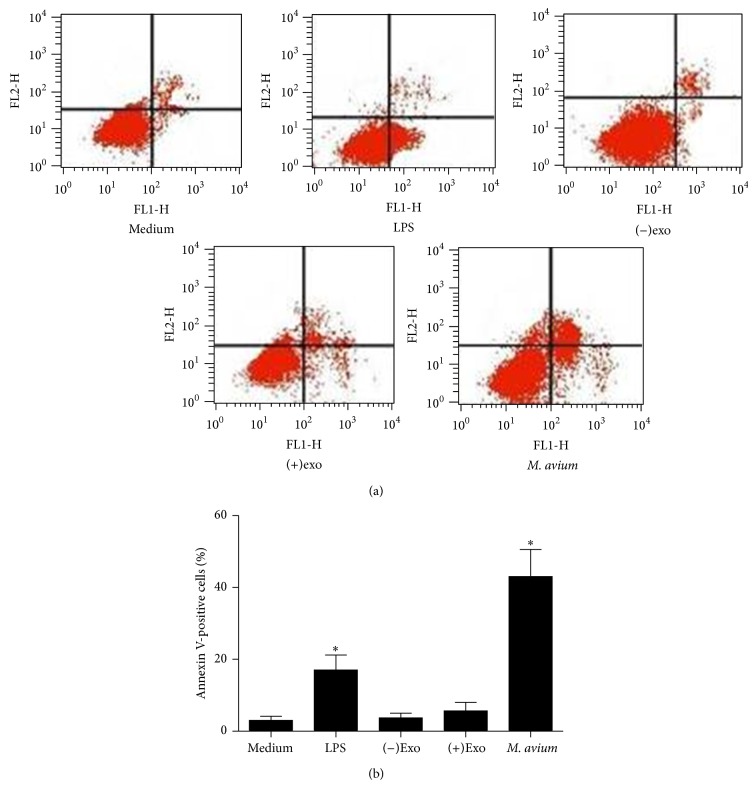
Apoptosis of macrophages treated with different stimuli as detected by flow cytometry. (a) Macrophages were treated with LPS (50 ng/mL), (−)exosomes (50 *μ*g/mL), (+)exosomes (50 *μ*g/mL), and* M. avium* (MOI of 10), respectively, for 24 h. (b) Quantification of apoptosis and necrosis, with asterisks indicating the values for which significant differences were observed. Results presented as mean ± SEM (*n* = 3); results are representative of three separate experiments, ^*^ *P* < 0.05 compared with medium alone.

*M. avium* phagocytized by macrophages can induce the apoptosis of host cells, which releases intracellular* M. avium* leading to the diffusion of* M. avium* in vivo and infecting surrounding macrophages. Macrophages treated with LPS, (−)exosomes, (+)exosomes, and* M. avium* were analyzed for cell membrane disruption and permeability with annexin V-FITC and PI staining by flow cytometry (Figure 6). As shown in the results,* M. avium* infection induced apoptosis or necrosis. However, (+)exosome treated macrophages did not have an increased level of apoptosis or necrosis, compared with the (−)exosomes, LPS, or medium alone treatments groups.

## 4. Discussion

Exosomes, 30–100 nm in diameter, are secreted vesicles derived from cell endosomal membrane system and have important implications in host biological functions. Exosomes secreted from* M. avium*-infected macrophages have been reported to contain many mycobacterial components including antigen 85-C, LpdC, PstS1, HspX, Mpt51, and Alanine [25], and they are proinflammatory [21]. Consistently, our results in the current study also demonstrated that (+)exosomes contained* M. avium*-derived proteins like ESAT-6, MPT63, SodA, MPT51, and antigen 85-C and these exosomes could induce macrophages to produce a panel of proinflammatory cytokines including IL-6, IL-8, IL-10, IFN-*γ*, and TNF-*α*. More importantly, our study for the first time revealed that (+)exosomes could trigger comparable immune responses as* M. avium* infection in the means of enhancing signaling protein expression on cell surface and proinflammatory cytokine production. However, unlike* M. avium* infection, (+)exosomes had no apparent effect on cell apoptosis and necrosis. Despite being beyond the scope of the current study, it is warranted to investigate whether such results can be translated to animal models or even clinical trials.

In general, (+)exosomes could induce similar immune responses as* M. avium* infection; however, slight difference was observed. For instance,* M. avium* infection could enhance the expression of CD32 and CD163, but not CD195 on cell surface, while (+)exosomes enhanced CD195 expression but not the other two CD molecules. In addition,* M. avium* but not (+)exosomes induced TGF-*β*1 level. CD32 negatively regulates IgG production by B cells [29]; CD163 is associated with a large range of inflammatory diseases including liver cirrhosis, type 2 diabetes, macrophage activation syndrome, Gaucher's disease, sepsis, HIV infection, rheumatoid arthritis, and Hodgkin lymphoma [30, 31], while CD195 functions as a chemokine receptor and is involved in recruitment of immunocytes, especially T cells, to site of infection [32]. Given the functions of these three CD molecules,* M. avium* infection induced CD32 upregulation might be associated with one of the mechanisms in which* M. avium* escapes from host immune response, and CD163 upregulation might be involved in* M. avium* infection causing disease manifestations, while the (+)exosomes induced CD195 expression could be related to enhanced host immune responses, especially T cell related responses. However, to fully understand the mechanisms as well as biological significance of the subtle difference between* M. avium* and (+)exosomes induced immune responses, further in-depth investigation is required.

It is noteworthy that although (+)exosomes could induce immune responses comparable to* M. avium* infection, they caused neither cell apoptosis nor necrosis. These characteristics make (+)exosomes strong candidate as vaccine. Albeit the exact mechanism that (+)exosomes do not induce apoptosis or necrosis is yet to be determined, some clues can be obtained in the results of our current study. In the cytokine quantification assay, we observed that only* M. avium* infection, but not (+)exosomes treatment, induced high levels of TGF-*β*1 expression. TGF-*β*1, a polypeptide member of the transforming growth factor beta superfamily, performs a variety of cellular functions, including control of cell growth, proliferation, differentiation, and apoptosis [33]. Dysregulation of TGF-*β* activation and signaling may result in apoptosis [34]. Consequently, TGF-*β*1-related singling pathway, although other pathways may also be involved, is likely to be responsible for* M. avium* induced cell apoptosis. Despite being beyond the scope of the current study, it is also interesting to determine which component(s) in* M. avium* can activate TGF-*β*1 pathway.

In conclusion, (+)exosomes could induce inflammatory immune responses comparable to* M. avium* infection but do not cause cell apoptosis. This suggests that exosomes would make a good vehicle for vaccine delivery.

## Figures and Tables

**Table 1:** Increased expression of CD40, CD80, CD81, CD86, CD195, and HLA-DR by macrophages treated with (+)exosomes, compared with other stimuli.

Treatment^a^	Surface molecule expression (median MFI, 25th and 75th percentile MFI)^b^								
	CD25	CD32	CD40	CD80	CD81	CD86	CD163	CD195	HLA-DR
Medium	19 (13, 29)	35 (29, 40)	99 (88, 109)	105 (98, 112)	109 (100, 121)	155 (144, 164)	98 (79, 111)	48 (41, 55)	100 (93, 113)
LPS	18 (11, 33)	38 (32, 44)	115^*^ (88, 128)	166 (156, 188)	154^*^ (139, 167)	223^*^ (209, 240)	97 (88, 104)	49 (40, 59)	106^*^ (100, 123)
(−)Exo	19 (13, 37)	40 (33, 50)	99 (75, 108)	105 (99, 123)	105 (95, 121)	150 (141, 162)	99 (90, 112)	67 (55, 77)	102 (90, 111)
(+)Exo	20 (17, 33)	54 (46, 89)	119^*^ (109, 131)	179^*^ (126, 211)	156^*^ (145, 173)	224^*^ (199, 245)	104 (85, 111)	91^*^ (83, 111)	132^*^ (109, 138)
*M. avium *	22 (18, 40)	82^*^ (70, 112)	122^*^ (115, 145)	201^*^ (190, 222)	179^*^ (157, 195)	279^*^ (258, 301)	111^*^ (88, 117)	68 (47, 70)	143^*^ (127, 155)

^a^Medium, incubated in medium only; LPS, activated by LPS; (−)exo, treated with (−)exosomes; (+)exo, treated with (+)exosomes; *M. avium*, infected with *M. avium*.

^b^Values are based on five independent experiments. MFIs of isotype controls were subtracted. Values in parentheses are the 25th and 75th percentile MFIs, respectively. ^*^ *P* < 0.05 compared with medium alone (Friedman test and Dunn's multiple comparison test).
